# TLR2 Regulates Neutrophil Recruitment and Cytokine Production with Minor Contributions from TLR9 during Hypersensitivity Pneumonitis

**DOI:** 10.1371/journal.pone.0073143

**Published:** 2013-08-30

**Authors:** Kelly Andrews, Hossam Abdelsamed, Ae-Kyung Yi, Mark A. Miller, Elizabeth A. Fitzpatrick

**Affiliations:** 1 Department of Microbiology, Immunology and Biochemistry, University of Tennessee Health Science Center, Memphis, Tennessee, United States of America; 2 Department of Pediatrics, University of Tennessee Health Science Center, Memphis, Tennessee, United States of America; University of Tübingen, Germany

## Abstract

Hypersensitivity pneumonitis (HP) is an interstitial lung disease that develops following repeated exposure to environmental antigens. The disease results in alveolitis, granuloma formation and may progress to a fibrotic chronic form, which is associated with significant morbidity and mortality. The severity of the disease correlates with a neutrophil rich influx and an IL-17 response. We used the *Saccharopolyspora* *rectivirgula* (SR) model of HP to determine whether Toll-like receptors (TLR) 2 and 9 cooperate in neutrophil recruitment and IL-17-associated cytokine production during the development of HP. Stimulation of bone marrow derived macrophages (BMDMs) from C57BL/6, MyD88^-/-^ and TLR2/9^-/-^ mice with SR demonstrate that SR is a strong inducer of neutrophil chemokines and growth factors. The cytokines induced by SR were MyD88-dependent and, of those, most were partially or completely dependent on TLRs 2 and 9. Following in vivo exposure to SR, CXCL2 production and neutrophil recruitment were reduced in TLR2^-/-^ and TLR2/9^-/-^ mice suggesting that the response was largely dependent on TLR2; however the reduction was greatest in the TLR2/9^-/-^ double knockout mice indicating TLR9 may also contribute to the response. There was a reduction in the levels of pro-inflammatory cytokines TNFα and IL-6 as well as CCL3 and CCL4 in the BALF from TLR2/9^-/-^ mice compared to WT and single knockout (SKO) mice exposed one time to SR. The decrease in neutrophil recruitment and TNFα production in the TLR2/9^-/-^ mice was maintained throughout 3 weeks of SR exposures in comparison to WT and SKO mice. Both TLRs 2 and 9 contributed to the Th17 response; there was a decrease in Th17 cells and IL-17 mRNA in the TLR2/9^-/-^ mice in comparison to the WT and SKO mice. Despite the effects on neutrophil recruitment and the IL-17 response, TLR2/9^-/-^ mice developed granuloma formation similarly to WT and SKO mice suggesting that there are additional mediators and pattern recognition receptors involved in the disease.

## Introduction

Hypersensitivity Pneumonitis (HP), or extrinsic allergic alveolitis, develops following repeated exposure to a wide variety of inhaled environmental antigens [1–5]. The disease is characterized by a lymphocytic alveolitis, noncaseating granulomas and, in some patients, develops into a chronic form which is associated with fibrosis and emphysema [1,3,6–8]. HP is a complex disease with components of Type III (antibody-mediated) and Type IV (cell-mediated) hypersensitivity reactions for which there are limited therapeutic options; the mainstay of treatment is avoidance of the inciting agent and corticosteroids which have limited effects on outcome. The environmental antigens that induce HP include organic dusts, vapors, fungi, bacteria, and molds as well as simple chemical compounds [6,9,10]. Exposure to these airborne antigens may occur in both occupational and residential settings and the different types of HP are frequently named after the occupation or activity that results in exposure to the inciting agent. Farmer’s Lung disease is one of the most common types of HP and is caused by repeated inhalation of the gram positive thermophile *Saccharopolyspora* *rectivirgula* (SR) which is commonly found in moldy hay [3].

The SR mouse model of Farmer’s Lung disease has been used to identify the pathogenic mechanisms leading to the disease [11,12]. Mice intranasally inoculated with SR for 3 days / week for 3 weeks develop a neutrophilic alveolitis that becomes more lymphocytic as exposures continue. The influx of neutrophils is accompanied by a cytokine response consisting of TNFα, IL-6, IL-17, IFNγ and CXCL2/MIP-2, macrophage and dendritic cell activation, and lymphocyte recruitment [13]. Granuloma formation is dependent on CD4^+^ T cells and both Th1 and Th17 cells are recruited to the lung although disease severity appears to correlate with the IL-17 response [14–16]. IL-17ra^-/-^ mice developed less severe inflammation following exposure to SR in comparison to WT mice [14]. IL-17-mediated pathology is frequently associated with neutrophil rich influxes and both IL-17 and neutrophils have been shown to correlate with the development of fibrosis in this model [17,18]. Identifying the pathways that trigger activation of neutrophil recruitment and the IL-17 response will provide important information on disease pathogenesis.

Activation of innate immune cells by microbial products occurs via stimulation through Toll-like receptors (TLRs; reviewed in 19). Individual TLRs have specificity for particular PAMPs; peptidoglycan, lipotechoic acids, and bacterial lipoproteins stimulate TLR2, flagellin stimulates TLR5, LPS and heat shock proteins stimulate TLR4, and unmethylated CpG motifs in microbial DNA or synthetic oligodeoxynucleotides stimulate TLR9. Activation of TLRs leads to the recruitment of adaptor proteins to the receptor complex and induction of a signaling cascade that result in the expression of numerous genes. With the exception of TLR3, all the TLRs use the MyD88 adaptor protein resulting in activation of the mitogen-activated protein kinase (MAPK) and NF-κB signaling pathways which lead to expression of genes involved in phagocytosis, cytokine and chemokine production, cell trafficking, survival and apoptosis. Our previous studies using the SR model of HP demonstrated that MyD88-dependent TLRs are necessary for the initial production of pro-inflammatory cytokines, neutrophil chemokines and subsequently neutrophil recruitment following acute SR exposure [20]. One of the MyD88-dependent TLRs involved in recognition of gram positive bacteria is TLR2 and our previous studies revealed that SR is recognized by TLR2 and can activate the TLR2 signaling pathway that leads to NF-κB activation in vitro. In addition, mice deficient in TLR2 had significantly reduced CXCL2/MIP-2 production accompanied by a partial reduction in neutrophil recruitment following one SR exposure. However, since TLR2 deficiency only affected CXCL2 production whereas MyD88^-/-^ mice had a complete absence of both proinflammatory cytokine production and neutrophil recruitment following SR exposure, there must be additional MyD88-dependent receptors involved [20]. TLR9 is an intracellular pattern recognition receptor (PRR) that is located within endosomes and phagolysosomes where it can interact with unmethylated CpG motifs in genomic DNA released from ingested microbes. Following phagocytosis of SR, CpG DNA motifs may stimulate TLR9 resulting in cytokine and chemokine gene expression thus contributing to the MyD88-dependent response.

In the Present Study We Examine the Extent to Which TLR2 and TLR9 Cooperate in Neutrophil Recruitment, IL-17-Associated Cytokine Production and Granuloma Formation during HP

## Materials and Methods

### Animals and *S. rectivirgula* exposure protocol

C57BL/6 female mice were purchased from Jackson Laboratories (Bar Harbor, ME) at 6 weeks of age. MyD88^-/-^, TLR2^-/-^, TLR9^-/-^, and TLR2/9^-/-^ mice on a C57BL/6 background were bred at UTHSC and were in at least the 5^th^ generation backcross. MyD88^-/-^ and TLR9^-/-^ were provided by Dr. S. Akira (Osaka University, Japan), TLR2^-/-^ mice were purchased from Jackson Lab and TLR2/9^-/-^ mice were generated by breeding TLR2^-/-^ and TLR9^-/-^ mice. All animals were housed in sterile micro-isolator cages with sterile food and water ad libitum and were maintained by the Division of Comparative Medicine at the University of Tennessee Health Science Center according to the guidelines of the Animal Welfare Act. All animal care procedures were performed according to protocols approved by the UTHSC animal care and use committee. The *S* *. rectivirgula* (strain designation A1313 – ATCC) was grown at 55°C in trypticase soy broth. The bacterial preparation was washed in endotoxin free distilled water 3 times followed by sonication and lyophilization. The lyophilized preparation was reconstituted with endotoxin free saline and mice inoculated intranasally with 50 µg three times / week for 3 weeks.

### Bronchoalveolar lavage (BAL) and lung cell isolation

BAL was performed by intratracheal injection of 1ml of PBS into the lungs with immediate vacuum aspiration. The amount of fluid recovered was routinely around 70%. Cells were recovered from the BAL fluid by centrifugation and counted using trypan blue dye exclusion and the BALF was frozen at -80°C until used in ELISA assays for cytokine and chemokine measurement. Lungs were perfused with phosphate-buffered saline (PBS) to remove blood and both lobes removed. Lung tissue was digested with collagenase (20 U/ml) and deoxyribonuclease I (40 µg/ml) for 45 minutes at 37°C. Cells were freed by disruption in a Stomacher tissue processor and then isolated by centrifugation on a discontinuous percoll gradient. Mononuclear cells were isolated at the 40/80% interface following density gradient centrifugation and used in flow cytometry or T cell stimulation assays.

### Bone marrow derived macrophage (BMDM) generation and stimulation

Bone marrow cells were harvested from the femurs and tibias of WT, TLR2^-/-^, TLR9^-/-^, TLR2/9^-/-^, and MyD88^-/-^ mice and cultured with media containing 30% L-929 culture supernatant as a source of M-CSF. The following day the non-adherent cells were harvested and plated with media containing M-CSF. On day 6-7 the adherent cells were harvested and the purity of the macrophage (CD11b^+^/F4/80^+^) preparation was determined by flow cytometry and was routinely between 80–85%. The BMDMs (5x10^5^ cells) were stimulated with SR (10 µg) for 24 hrs and cell culture supernatants were harvested and stored at -80°C for cytokine and chemokine analysis by ELISA.

#### Flow cytometry

Flow cytometry was performed on isolated BAL and lung cells using fluorochrome-conjugated antibodies to CD11b, Gr1, F4/80, CD45, CD4, CD8, and βTcR (BD Biosciences, San Jose, CA or ebiosciences, San Diego, CA). For intracellular cytokine staining, lung cells were incubated with SR lysate (4µg) for 3 h with an additional 3 h in the presence of GolgiStop (BD Biosciences). The cells were incubated with antibodies to CD45, CD4, and βTcR to identify CD4^+^ T cells and then fixed and permeabilized with 1% saponin followed by incubation with IL-17A, IL-17F, IL-10, or isotype control antibodies. The SR lysate used in the assay was generated using the Y-PER reagent (Pierce) following manufacturer’s instructions. A minimum of 50,000 events / sample was collected on a BD Biosciences LSRII cytometer (BD Biosciences, San Jose, CA). Expression of cell surface markers and intracellular cytokines was analyzed using DIVA software.

### ELISA

Cytokines present in unconcentrated BALF or culture supernatants were measured by ELISA according to manufacturer’s instructions. Cytokine standards ranging from 15.6 pg/ml to 4,000 pg/ml were prepared to determine the concentration of cytokine in the samples. For data analysis, a curve fit was applied to the standards and the sample concentrations extrapolated from the standard curve using four-parameter logistic software (SoftMax Pro, Sunnyvale, CA). For BMDM cell culture supernatants, a Milliplex 32-plex bead based ELISA assay (Millipore) was performed according to the manufacturer’s instructions.

### PCR

Total RNA was extracted from the upper right lobe of lung from individual mice using Trizol. Contaminating genomic DNA was removed by treatment with DNA-free (Ambion, Austin, TX) according to manufacturer’s directions. One µg of RNA was reverse transcribed into cDNA with the Roche Transcriptor First Strand cDNA synthesis kit (Roche Applied Science, Indianapolis, IN). Real time PCR was performed with gene specific primers using a LightCycler 480 real time PCR thermal cycler. All primers and probes were chosen using the online software Universal Probe Library. Data are normalized for HPRT and plotted as fold induction over unexposed control mice.

### Histology

The left lobe of lung was removed and fixed with neutral buffered formalin and embedded in paraffin. Eight micron sections were cut and stained with Hematoxylin and Eosin (H&E) to analyze granuloma formation.

### Statistics

Data were analyzed using one-way ANOVA with Tukey-Kramer post hoc test using GraphPad Prism statistical software (GraphPad Software, San Diego, CA). Differences were considered significant at *p* values of less than 0.05. Results are expressed as mean ± S.D.

## Results

### TLRs 2 and 9 contribute to cytokine production by BMDMs following SR stimulation

**Figure 1:**
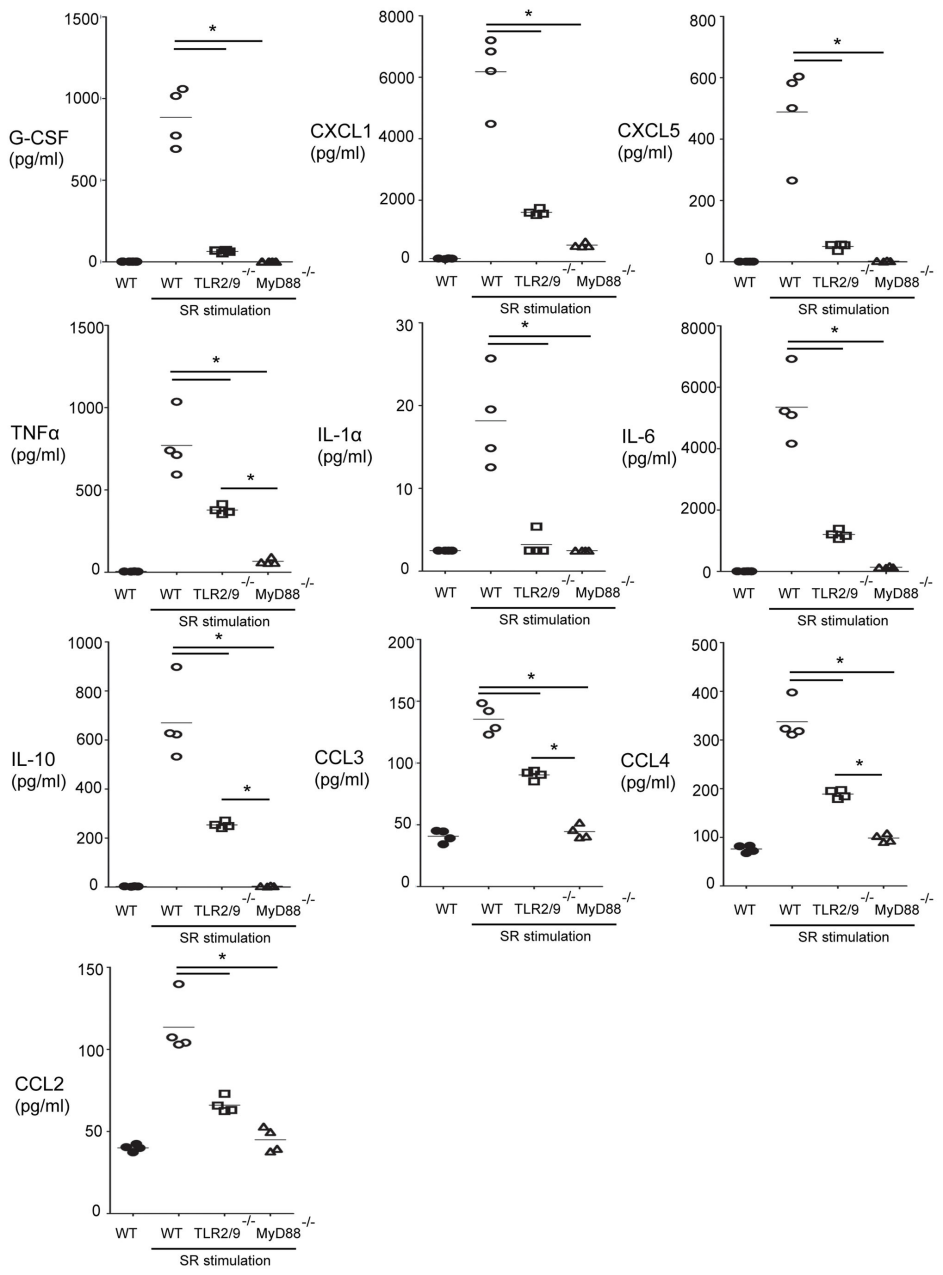
Cytokine production by WT and mutant BMDMs following SR stimulation. WT, TLR2/9^-/-^ and MyD88^-/-^ BMDMs were cultured with or without SR (0.05 mg/ml) for 24 h and cell culture supernatants analyzed for cytokine production by a 32-plex bead based ELISA. The results are expressed as the mean ± SD (*n* = 4 per group). *p < 0.05.

To determine whether SR induced cytokine or chemokine production through MyD88 or TLRs 2 and 9, we generated BMDMs from WT, TLR2/9^-/-^ or MyD88^-/-^ mice and stimulated them in vitro with SR. The cell culture supernatants were harvested following an overnight incubation and cytokine or chemokine production measured in a bead based ELISA. The results demonstrate that SR stimulates numerous cytokines in a MyD88-dependent manner including G-CSF, CXCL1/KC, CXCL5/LIX, TNFαIL-1αIL-6, IL-10, CCL3/MIP-1α, CCL4/MIP-1β, and CCL2/MCP-1 (Figure 1). The combined deficiency of TLRs 2 and 9 led to complete inhibition of G-CSF, CXCL5, and IL-1α production suggesting that they are the only MyD88-dependent TLRs contributing to the production of those cytokines and chemokines following SR stimulation. However, compared to WT cells, CXCL1, TNFα, IL-6, IL-10, CCL3, CCL4, and CCL2 were significantly reduced in TLR2/9^-/-^ BMDMs but not to the level of the MyD88^-/-^ cells suggesting the possibility that an additional MyD88-dependent receptor(s) is contributing to their production. Production of CCL5/RANTES and CXCL10/IP-10 by SR stimulated BMDMs was completely independent of MyD88 and CXCL2 was partially dependent on MyD88 suggesting SR also stimulates through MyD88-independent PRRs for chemokine production (data not shown). SR did not stimulate production of eotaxin, GM-CSF, IFNγ, IL-2, IL-3, IL-4, IL-5, IL-7, IL-9, IL-13, IL-15, IL-17 or LIF in BMDMs from WT, TLR2/9^-/-^, or MyD88^-/-^ mice. These results suggest that TLRs 2 and 9 contribute significantly to stimulation of proinflammatory cytokines necessary for development of HP.

### TLRs 2 and 9 contribute to neutrophil chemokine production and recruitment into the airways following one exposure to SR

**Figure 2:**
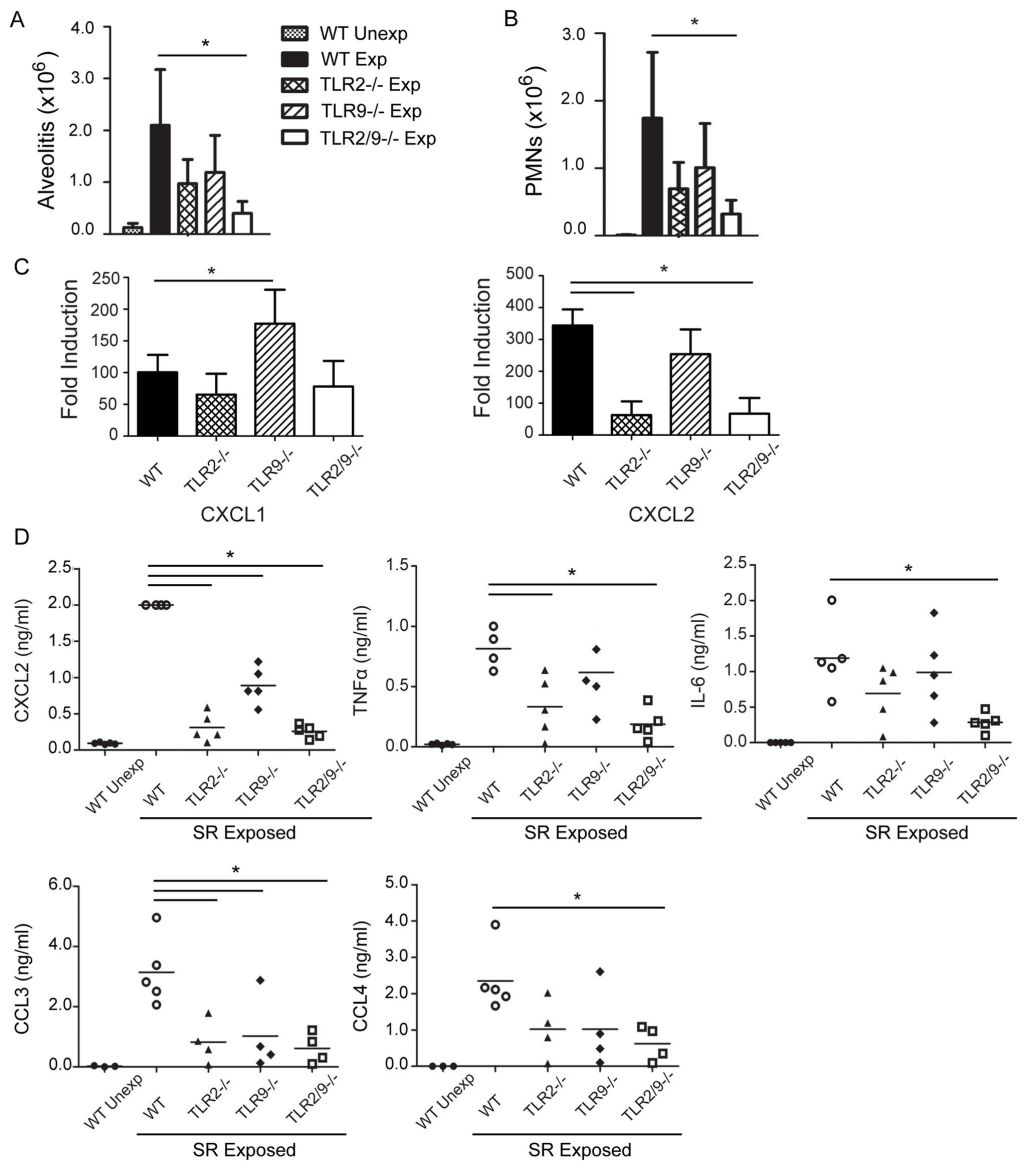
Contribution of TLRs 2 and 9 to inflammation following a single SR exposure. WT, TLR2^-/-^, TLR9^-/-^, and TLR2/9^-/-^ mice were exposed to SR and analyzed 6 h after one exposure. (A) BAL was performed and alveolitis determined using trypan blue dye exclusion. (B) Cells recovered from the BAL fluid were incubated with antibody to CD11b and Gr1 and analyzed by flow cytometry to determine the number of neutrophils (CD11b^+^/Gr1^high^) in each group. (C) Expression of mRNA for cytokines was determined by qRT-PCR on RNA isolated from individual lung lobes (*n* = 3-5 mice per group) and is expressed as fold induction over WT unexposed mice. The data represent mean ± SD of duplicate samples and significance was determined using one-way ANOVA with Tukey’ post-hoc test (*p < 0.05 compared to WT / SR exposed mice). (D) The cell-free BAL fluid was analyzed for CXCL2, TNFα and IL-6 by ELISA and CCL3 and CCL4 by Milliplex bead based ELISA. Data represent the mean ± SD (n = 5 per group) significance was determined using one-way ANOVA with Tukey’ post-hoc test (*p < 0.05 compared to WT SR exposed mice).

Our previous studies demonstrated that neutrophil recruitment was dependent on MyD88 signaling pathways and that TLR2 was one of the MyD88-dependent PRRs that contributed to CXCL2 production following a single SR exposure [20]. The results with the BMDMs also support that SR is a strong inducer of neutrophil chemokines such as CXCL5, CXCL1, CXCL2 and the neutrophil growth factor G-CSF and that TLRs 2 and 9 contribute to production of these cytokines. To examine the combined role of TLRs 2 and 9 in neutrophil recruitment following in vivo exposure to SR we exposed WT, TLR2^-/-^, TLR9^-/-^, and TLR2/9^-/-^ mice to SR and measured the recruitment of neutrophils into the airways (Figure 2). Analysis of the BAL from individual mice demonstrated that following a single exposure to SR, there is an increase in alveolitis in the WT mice which is significantly reduced in the TLR2/9^-/-^ mice. Although the TLR2^-/-^ mice, (and to a lesser extent the TLR9^-/-^ mice), had reduced alveolitis compared to the WT mice, the differences were not statistically significant (Figure 2A). The reduction in alveolitis in the TLR2/9^-/-^ mice correlated with the decrease in neutrophil recruitment that was observed compared to WT mice (Figure 2B). Both TLRs 2 and 9 appear to contribute to neutrophil recruitment following a single SR exposure since a significant reduction in neutrophils was only observed in the TLR2/9^-/-^ mice. The decrease in neutrophil recruitment was likely due to a decrease in chemokine production and not an intrinsic defect in TLR2/9^-/-^ neutrophil migration since neutrophils in the TLR2/9^-/-^ mice migrated into the peritoneum in response to an IP injection of casein (data not shown). The results of our studies with BMDMs suggest that TLRs 2 and 9 contribute to the production of the neutrophil chemokines CXCL1 and CXCL2; therefore we measured expression of these chemokines in the lungs of WT, TLR2^-/-^, TLR9^-/-^, and TLR2/9^-/-^ mice following a single SR exposure. Quantitative RT-PCR analysis revealed that there is a significant decrease in CXCL2 mRNA in the TLR2^-/-^ and TLR2/9^-/-^ mice; however there was no decrease in CXCL1 mRNA in any of the mutant mice (Figure 2C). The decrease in CXCL2 mRNA was reflected in the BALF; TLR2^-/-^, TLR9^-/-^ and TLR2/9^-/-^ mice had a significant decrease in CXCL2 in the BALF following SR exposure (Figure 2D). TLR2 appeared to be important for TNFα and IL-6 production (which may contribute to neutrophil recruitment) in vivo since they were reduced in the BALF of TLR2^-/-^ and TLR2/9^-/-^ mice exposed to SR in comparison to the WT and TLR9^-/-^ mice. We also observed a decrease in the chemokines CCL3 and CCL4 in the TLR2/9^-/-^ mice compared to the WT mice suggesting that TLRs 2 and 9 may also be important for recruitment of monocytes and lymphocytes following a single SR exposure. Although G-CSF was completely dependent on TLRs 2 and 9 following in vitro SR stimulation of BMDMs, we did not find a significant decrease in G-CSF in the BALF of exposed mice (data not shown). This suggests that lack of the neutrophil growth factor was not the cause of decreased neutrophil recruitment in the mice and that in vivo G-CSF produced in response to SR exposure is not dependent on TLR 2 or 9. In addition, we did not see an increase in Annexin V staining of neutrophils in the BALF of exposed TLR2/9^-/-^ mice suggesting that increased apoptosis is not playing a role in the decrease in neutrophil frequency in the BALF of these mice (data not shown). Altogether, these results suggest that following a single SR exposure, neutrophil chemokine production and recruitment is largely dependent on TLR2 and any role for TLR9 becomes apparent only in the absence of TLR2.

### Neutrophil recruitment and chemokine production are largely dependent on TLR2 following repeated SR exposures

**Figure 3:**
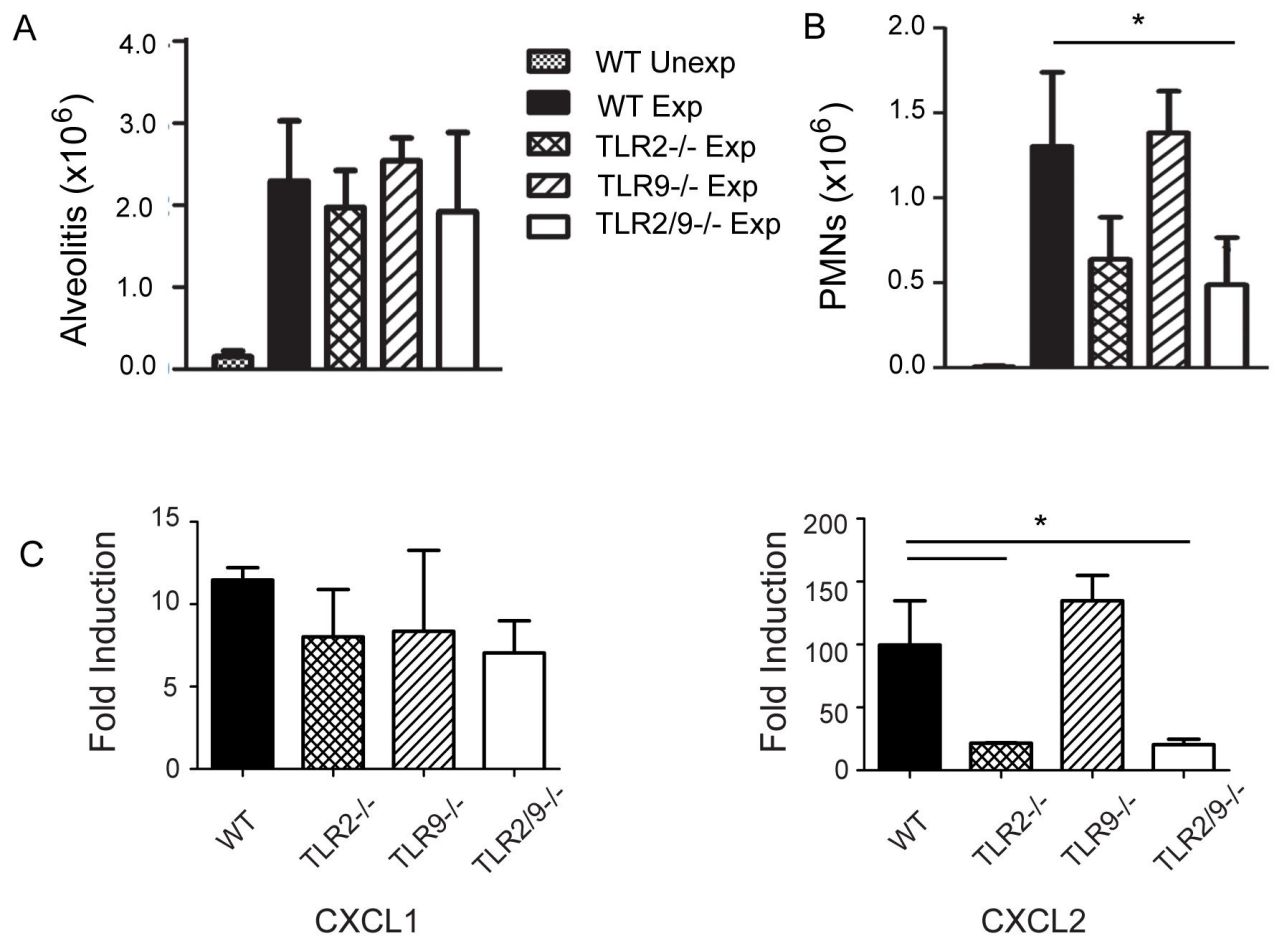
Neutrophil recruitment following repeated SR exposures is primarily dependent on TLR2. WT, TLR2^-/-^, TLR9^-/-^, and TLR2/9^-/-^ mice were exposed to SR 3 times / week for 3 weeks and analyzed 1 day after the last exposure. (A) BAL was performed and alveolitis determined using trypan blue dye exclusion. (B) Cells recovered from the BAL fluid were incubated with antibody to CD11b and Gr1 and analyzed by flow cytometry to determine the number of neutrophils (CD11b^+^/Gr1^high^) in each group. (C) Expression of mRNA for cytokines was determined by qRT-PCR on RNA isolated from individual lung lobes (*n* = 3-5 mice per group) and is expressed as fold induction over WT unexposed mice. The data represent mean ± SD of duplicate samples and significance was determined using one-way ANOVA with Tukey’ post-hoc test (*p < 0.05 compared to WT SR exposed mice).

To determine whether TLRs 2 and 9 also control neutrophil recruitment throughout 3 weeks of repeated exposures (at which time granulomas develop) we exposed WT, TLR2^-/-^, TLR9^-/-^, and TLR2/9^-/-^ mice to SR 3 times / week for 3 weeks and performed BAL one day after the last exposure. The results demonstrate that there is no significant reduction in alveolitis in the TLR2/9^-/-^ mice compared to the WT mice at day 18 (Figure 3A). However, the composition of the alveolitis in TLR2/9^-/-^ mice is different with a significant reduction of neutrophil frequency compared to the WT group (Figure 3B). The decrease in neutrophil recruitment to the airways appeared to be compensated for by an increase in CD11b^+^/Gr1^-^/CD11c^-^/F4/80^-^ cells. The percentages of CD4^+^ or CD8^+^ T cells and B cells varied but were not significantly different between the WT and mutant mice (data not shown). Likewise, the percentage of activated (CD69^+^) γδT cells also varied but was not different between the WT and mutant mice (data not shown). The decrease in neutrophil recruitment in the TLR2/9^-/-^ mice correlated with a significant decrease in CXCL2 mRNA in both the TLR2^-/-^ and TLR2/9^-/-^ mice compared to the WT mice (Figure 3C). However, CXCL1 mRNA was not significantly reduced between the WT and mutant mice suggesting that TLRs 2 and 9 are not responsible for its production. These results demonstrated that neutrophil recruitment and CXCL2 production following repeated SR exposures is largely dependent on TLR2.

### TLRs 2 and 9 cooperate in cytokine production following repeated SR exposures

**Figure 4:**
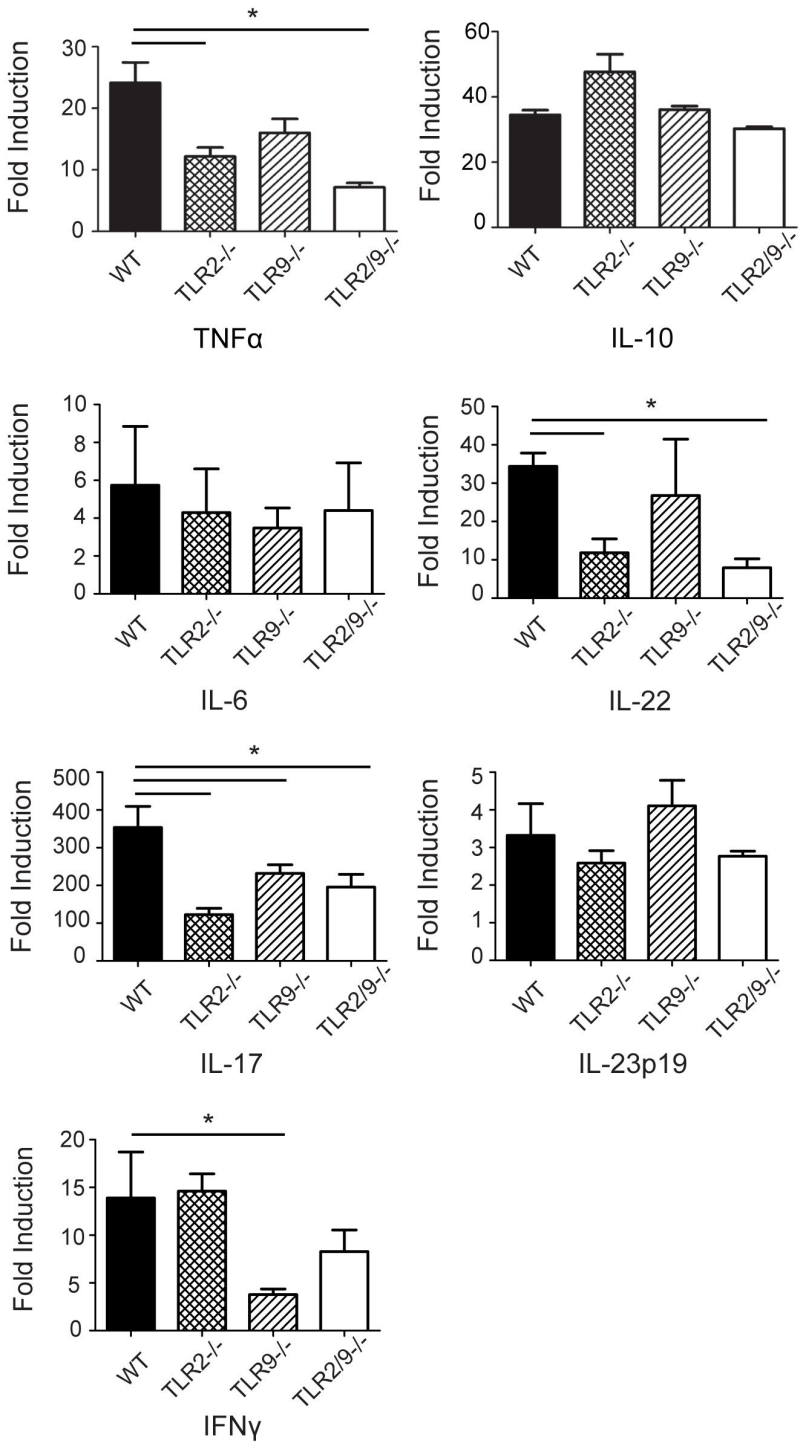
TLRs 2 and 9 contribute to Th1- and Th17-associated cytokine production following repeated SR exposures. WT, TLR2^-/-^, TLR9^-/-^ and TLR2/9^-/-^ mice were exposed to SR 3 times / week for 3 weeks and analyzed 1 day after the last exposure. Lungs were removed and qRT-PCR of RNA isolated from individual lung lobes was performed to measure the expression of individual cytokines using specific primers. The results were normalized to the housekeeping gene HPRT and expressed as fold induction over unexposed mice. Significance was determined using one-way ANOVA with Tukey’ post-hoc test (*p < 0.05 compared to WT SR exposed mice).

The production of pro-inflammatory cytokines and IL-17 associated cytokines is critical to development of HP. TNFα is required for disease development, IL-10 plays an inhibitory role and IL-6 is necessary for the development of the Th17 response [14–16]. To determine whether cytokine production was dependent on TLRs 2 or 9 following repeated SR exposures, we used qRT-PCR to measure expression of cytokine mRNA in the lungs of mice exposed to SR for 3 weeks (Figure 4). TNFα mRNA, which was decreased during acute exposure in TLR2/9^-/-^ mice, was significantly decreased in the lungs of TLR2^-/-^ and TLR2/9^-/-^ mice compared to the WT or TLR9^-/-^ mice following 3 weeks of exposure. Expression of IL-17 and IL-22, both of which contribute to disease severity, were also significantly reduced in the TLR2^-/-^ and TLR2/9^-/-^ mice compared to the WT or TLR9^-/-^ mice; however IL-6 and IL-23p19 expression were not reduced in the TLR2/9^-/-^ mice. We did not detect an increase of IL-10 mRNA in the lungs of TLR2/9^-/-^ mice exposed for 3 weeks, indicating that a decrease in inflammation is not related to expression of IL-10. Expression of the Th1 cytokine IFNγ was significantly reduced in TLR9^-/-^ mice in comparison to WT, TLR2^-/-^ and TLR2/9^-/-^ mice following SR exposure suggesting that TLR9 is important in IFNγ expression. These results suggest that TNFαIL-17and IL-22 production is largely mediated by TLR2 stimulation following repeated exposure with SR.

### TLRs 2 and 9 are not required for lymphocyte recruitment or granuloma formation

**Figure 5:**
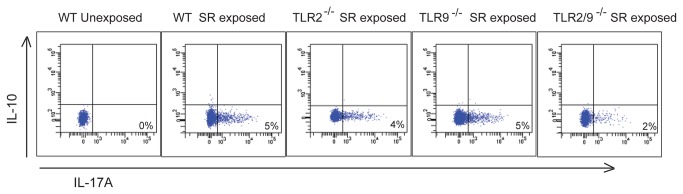
TLRs 2 and 9 cooperate in generation of the Th17 response following repeated SR exposures. WT, TLR2^-/-^, TLR9^-/-^, and TLR2/9^-/-^ mice were exposed to SR 3 times / week for 3 weeks and analyzed 4 days after the last exposure. Lung cells were stimulated with SR lysate or media alone for 6 hrs and intracellular cytokine staining performed to measure expression of IL-17A and IL-10 by CD4^+^/βTcR^+^ cells. Representative dot plots are shown (n = 3; repeated 2 times) Mean ± SD of CD4^+^/IL-17A^+^ WT unexposed = 0 ± 0%; CD4^+^/IL-17A^+^ WT exposed = 6.7 ± 2.2%; CD4^+^/IL-17A^+^ TLR2^-/-^ exposed = 4.8 ± 0.8%; CD4^+^/IL-17A^+^ TLR9^-/-^ exposed = 5.4 ± 1.0%; CD4^+^/IL-17A^+^ TLR2/9^-/-^ exposed = *2.5 ± 1.4%. Significance was determined using one-way ANOVA (*p < 0.05 compared to WT SR exposed mice).

With repeated SR exposures an adaptive immune response develops which is associated with T cell dependent granuloma formation. To determine whether TLRs 2 or 9 were necessary for lymphocyte recruitment into the lung, we exposed WT, TLR2^-/-^, TLR9^-/-^, and TLR2/9^-/-^ mice to SR 3 times / week for 3 weeks and performed flow cytometric analysis of immune cells within the interstitial lung tissue. The results revealed that the percentage of CD4^+^ or CD8^+^ T cells and B cells did not significantly differ between the WT and mutant groups of mice (data not shown). However, there was a significant decrease in the frequency of activated CD4^+^ T cells between WT and TLR2^-/-^ mice (WT CD4^+^/CD69^+^ = 56 ± 6.6% vs TLR2^-/-^ CD4^+^/CD69^+^ = 42 ± 0.9%; *p* < 0.05) and between WT and TLR2/9^-/-^ mice (WT CD4^+^/CD69^+^ = 56 ± 6.6% vs TLR2/9^-/-^ CD4^+^/CD69^+^ = 41 ± 2.9%; *p* < 0.05). We measured the percentage of Th17 cells recruited into the lungs of the TLR2/9^-/-^ mice using intracellular cytokine staining (Figure 5). The results demonstrate that following stimulation with SR lysate, 5% of CD4^+^ T cells from the lungs of WT, TLR2^-/-^ or TLR9^-/-^ mice responded with IL-17 production. The isoform of IL-17 was IL-17A; there were negligible amounts of IL-17F produced following stimulation (data not shown). However, in the absence of TLRs 2 and 9 only about 2% of the CD4^+^ T cells responded to SR stimulation. There was no increase in IL-10 expressing T cell frequency, and the decrease in Th17 cell frequency observed in the TLR2/9^-/-^ mice was not accompanied by an increase in Th1 cells in those mice (data not shown).

**Figure 6:**
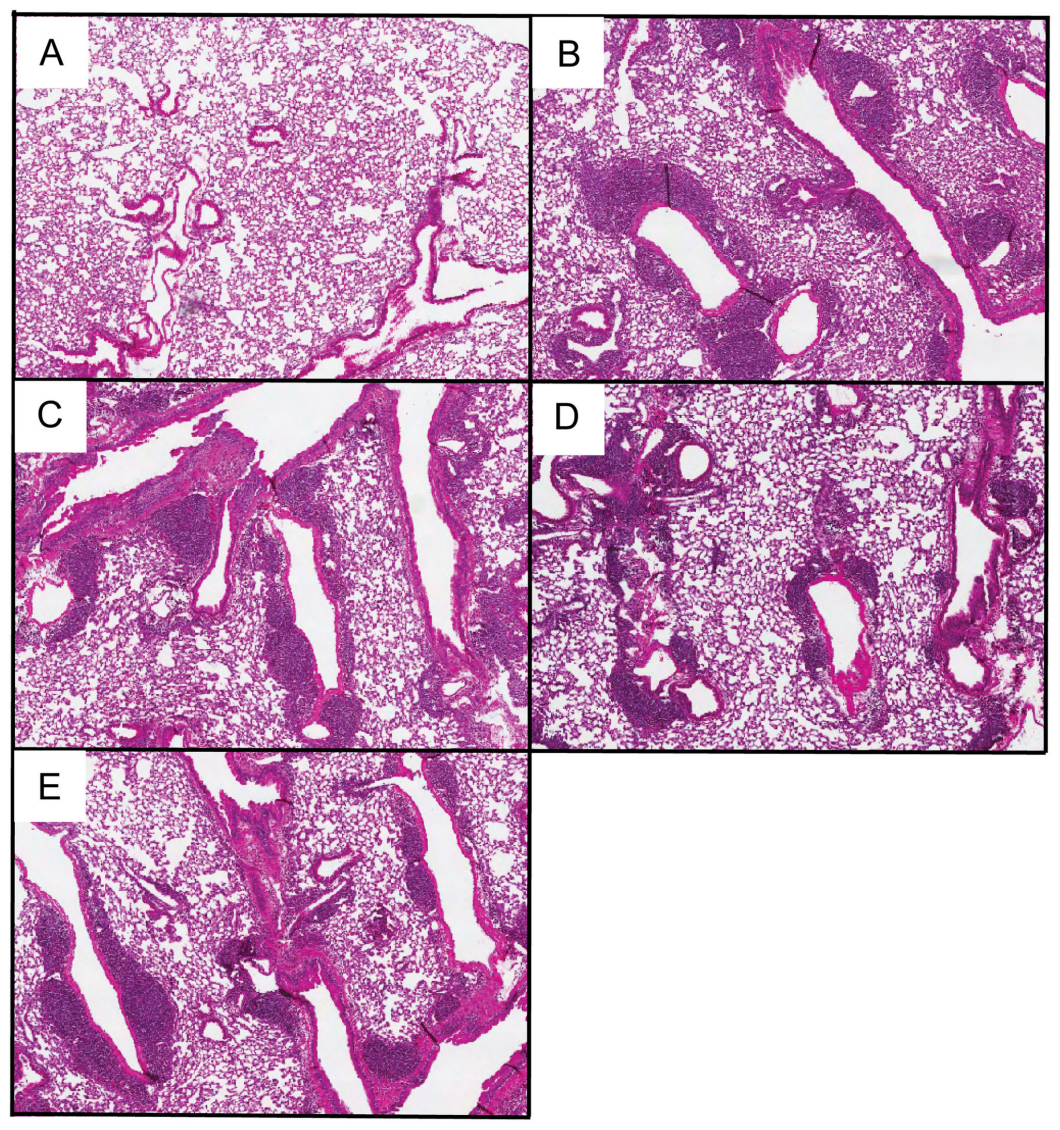
Granuloma development is not dependent on TLRs 2 and 9. WT, TLR2^-/-^, TLR9^-/-^, and TLR2/9^-/-^ mice were exposed to SR 3 times / week for 3 weeks and analyzed 3 days after the last exposure. Representative H&E stained lung sections from unexposed WT mice (A), WT mice (B), TLR2^-/-^ mice (C), TLR9^-/-^ mice (D) and TLR2/9^-/-^ mice (E) exposed to SR (Original magnification x4).

To determine whether the decrease in neutrophils, IL-17 and activated CD4^+^ T cells in the lungs of TLR2/9^-/-^ mice resulted in reduced development of granulomas, we examined H&E stained lung sections from WT and mutant mice that had been exposed to SR for 3 weeks (Figure 6). Wild type mice exposed to SR displayed peribronchiolar areas of granuloma formation while unexposed mice exhibited normal lung architecture (Figure 6A and B). The TLR2^-/-^, TLR9^-/-^ and TLR2/9^-/-^ mice exposed to SR all demonstrated granuloma formation (Figure 6C, D and E) suggesting that additional PRRs must be involved in granuloma formation in HP.

## Discussion

The results from our studies suggest that TLR 2 is largely responsible for neutrophil recruitment and TNFα, IL-17 and IL-22 production following SR exposure although there appears to be some contribution from TLR9. Neutrophils play an important role in the development of lung damage throughout the different stages of HP. They are the first cell population recruited into the lungs following SR exposure and they release numerous cytokines such as TNFα and IFNγ that contribute to the initiation of disease [21,22]. There is a positive correlation between lung neutrophils and lung fibrosis in patients with chronic HP and an increase in gelatinase B and collagenase-2 activity that may contribute to the fibrotic response [17,23]. The results from the in vitro BMDM studies demonstrated that SR is a strong inducer of neutrophil chemokines and growth factors (CXCL1, CXCL2, CXCL5, and G-CSF), a finding that correlates with patient studies demonstrating high levels of IL-8 in BALF [24]. With the exception of CXCL2, the neutrophil chemokines produced by BMDMs were completely dependent on MyD88 and the combined deficiency of TLRs 2 and 9 significantly reduced their production. The results of our in vivo studies using TLR2^-/-^ or TLR9^-/-^ SKO mice and TLR2/9^-/-^ mice exposed to SR demonstrated the importance of TLR2 in neutrophil recruitment and CXCL2 production following repeated SR exposures. There was a significant decrease in CXCL2 protein in the BALF of TLR2^-/-^, TLR9^-/-^ and TLR2/9^-/-^ mice exposed to SR compared to WT mice following a single SR exposure. Following repeated exposures only the TLR2^-/-^ and TLR2/9^-/-^ mice had reductions in CXCL2 mRNA and neutrophils in the lung suggesting that the contribution from TLR9 is negligible at this time point. Our in vitro studies suggested that TLRs 2 and 9 were required for G-CSF and CXCL1 production; however we did not observe a decrease in G-CSF in BALF or CXCL1 mRNA from SR exposed TLR2/9^-/-^ mice compared to the WT mice suggesting that these cytokines are induced independently of those TLRs in vivo. The residual neutrophil recruitment observed in the lungs of the TLR2/9^-/-^ mice exposed to SR may be in response to the CXCL1 that was present.

TLR stimulation by SR is also critical for production of proinflammatory cytokines during development of HP. Stimulation of BMDMs from WT, TLR2/9^-/-^ or MyD88^-/-^ mice indicated that SR stimulates production of proinflammatory cytokines such as TNFα, IL-1α and IL-6 but not cytokines commonly associated with a Th2 response. This is consistent with the disease being a non-IgE mediated hypersensitivity disorder and with murine studies demonstrating that a Th2 response may be protective [25–27]. The majority of the tested cytokines stimulated by SR were completely dependent on MyD88 signaling, revealing the importance of MyD88-dependent TLRs or IL-1R/IL-18R in the host response to SR. All of the MyD88-dependent cytokines induced by SR were partially or completely dependent on TLRs 2 and 9 suggesting that these are the major TLRs involved in the response. However, there is clearly another MyD88-dependent receptor that contributes to the response since cytokines such as TNFα and IL-6 were only partially inhibited in the TLR2/9 double deficient cells. Expression of three chemokines that are important for recruitment of macrophages and lymphocytes to the lung (CCL5, CXCL10 and CXCL2) was mostly independent of MyD88. This finding suggested that induction of these chemokines involves signaling via additional PRRs. SR is a gram positive microbe with a type IV cell wall containing meso-diaminopimelic acid which may stimulate through intracellular PRRs such as NOD1 that have been found to contribute to responses mounted against extracellular bacteria [28,29]. Therefore the host response to SR is likely to involve the activation of multiple TLRs and other PRRs resulting in the development of the inflammatory response. Following in vivo exposure of WT and mutant mice to SR, our results suggested that TLR2 was more important in TNFα production since it was significantly reduced in TLR2^-/-^ and TLR2/9^-/-^ at day 1 and 18 of exposures. However since the reduction in TNFα was greater in the TLR2/9^-/-^ mice it is likely that TLR9 is also contributing to the response. Since neutrophils are important sources of TNFα, it is possible that the decrease in neutrophil recruitment is responsible for the decrease in TNFα in the TLR2/9^-/-^ mice. IL-6 was decreased in the BALF of TLR2^-/-^ mice, but it was only in the TLR2/9^-/-^ mice that the reduction became significant suggesting that both TLRs 2 and 9 contribute to its production following a single SR exposure. Following repeated SR exposures the level of IL-6 mRNA in the TLR2/9^-/-^ mice was not different than WT or SKO mice suggesting that other factors contribute to its production at later time points. In contrast to TNFα and IL-6, there was no reduction in IL-10 mRNA in the WT or mutant mice at day 18 of SR exposure, so it appears that IL-10 induction is independent of both TLR2 and TLR9 at this time point. These results also suggest that any decrease in inflammation in the TLR2/9^-/-^ mice is not due to an increase in IL-10 production. Because the cell populations and architecture of the lung are more complex than that of BMDMs cultured in vitro, and because there are likely differences in TLR2 and TLR9 expression levels, we were not surprised when the results of the in vivo and in vitro experiments were not always in agreement. The contribution of TLR9 to cytokine production following SR exposure may be more apparent when analyzing homogenous cell populations rather than BALF or whole lung homogenates.

Patients with HP exhibit a lymphocytic alveolitis consisting of CD4^+^ and CD8^+^ T cells, and microarray studies performed on lung biopsies from patients indicate an increase in expression of genes associated with inflammation and T cell activation [30–32]. Our studies suggest that lymphocyte activation is also primarily regulated by TLR2 with modest cooperation from TLR9. Disease severity is associated with IL-17 (which is also produced by γδT cells); in murine models inhibition of IL-17 production results in a decrease in inflammation [15,33]. Fong et al [34] identified TLR6 as being important for expression of IL-17A in this model. TLR6 forms a heterodimer with TLR2 and in our study expression of IL-17 mRNA was decreased in TLR2^-/-^, TLR9^-/-^ and TLR2/9^-/-^ mice compared to WT mice suggesting that both TLRs 2 and 9 also contribute to its production. There may be several factors involved in the decrease in IL-17 mRNA. Numerous cells can produce IL-17 in addition to CD4^+^ T cells such as NK cells and γδT cells. It has been reported that γδT cells express TLR2 and can be stimulated by TLR2 ligands to produce IL-17 and this may contribute to the decrease in IL-17 in the TLR2^-/-^ and TLR2/9^-/-^ mice [35]. We only observed a modest decrease in the percentage of Th17 cells in the TLR2/9^-/-^ mice exposed to SR. The decrease in Th17 cells does not appear to be due to a decrease in IL-23 which is necessary for survival of the Th17 cell lineage and maintaining IL-17 production since we did not observe a decrease in IL-23p19 mRNA in the TLR2/9^-/-^ mice. Although the decrease in Th17 cells could be complimented by an increase in other T cell subtypes, we could not detect Th1 cells in the TLR2/9^-/-^ mice and RT-PCR did not reveal an increase in IL-4 mRNA suggesting that there was no switch in the T cell response. Expression of IL-22 was dependent on TLR2 since both TLR2^-/-^ and TLR2/9^-/-^ mice had reduced IL-22 mRNA following repeated SR exposures. IL-22 has been suggested to play a protective role in the disease by inhibiting CD4^+^ T cell migration and collagen deposition [36] and the decrease in IL-22 in the TLR2/9^-/-^ mice may explain the development of granulomas despite decreased T cell activation and TNFα production in these mice. Despite the decrease in activated lymphocytes, TNFα and IL-17, and neutrophil recruitment in the TLR2/9^-/-^ mice, there was no significant difference in granuloma formation compared to the WT mice following 3 weeks of SR exposure. This suggests that additional PRRs are involved in the granulomatous response; these may include non-TLR family members as well as TLRs that respond to danger associated molecular patterns (DAMPs) generated during the inflammatory response.

Overall our results suggest that TLR2 is the major TLR involved in numerous aspects of disease pathogenesis during HP such as neutrophil recruitment, lymphocyte activation and Th17 associated cytokine production. The contributions from TLR9 were less significant and only became apparent in the absence of TLR2 which suggest that some cooperation between TLRs 2 and 9 contribute to the development of HP.
